# Plant‐Based Diet Quality and Gastric Cancer Risk: A Case–Control Study in High‐Risk Regions of Fujian Province, China

**DOI:** 10.1002/fsn3.71756

**Published:** 2026-04-13

**Authors:** Xinyu Chen, Qingying Wang, Xuehui Zhang, Fengqin Zou, Yaqing Wu, Wanling Zeng, Sifang Li, Yulan Lin

**Affiliations:** ^1^ Department of Epidemiology and Health Statistics, Fujian Provincial Key Laboratory of Environment Factors and Cancer, School of Public Health Fujian Medical University Fuzhou China; ^2^ Department of Health Law and Humanistic Management, School of Health Management Fujian Medical University Fuzhou China

**Keywords:** case–control study, dietary quality, gastric cancer, plant‐based dietary index

## Abstract

Plant‐based diets have been increasingly associated with cancer prevention, but the distinct impacts of healthy and unhealthy plant‐based foods on gastric cancer (GC) risk remain uncertain. This study aimed to investigate the associations of the overall plant‐based dietary index (PDI), the healthy PDI (hPDI), and the unhealthy PDI (uPDI) with GC risk. A sex‐matched case–control study was carried out in Fujian, China, between July 2023 and November 2024, including 336 newly diagnosed GC patients and 336 community controls. Dietary intake was evaluated using a validated food frequency questionnaire. In total, 672 participants (56.5% male) were analyzed, with cases being older than controls (mean age 56.76 vs. 53.86 years, *p* < 0.001). Higher PDI was associated with a lower risk of GC (Q4 vs. Q1: OR = 0.55, 95% CI: 0.35–0.84, *p* for trend = 0.001). For hPDI, the highest versus lowest quartile comparison suggested an inverse association (OR = 0.63, 95% CI: 0.41–0.96), although the trend test was marginally significant (*p* for trend = 0.069). Each 1‐SD increase in PDI was associated with a 26% lower risk of GC (OR = 0.74, 95% CI: 0.63–0.87, *p* < 0.001). Conversely, higher uPDI was associated with a markedly increased risk (OR = 2.42, 95% CI: 1.55–3.78, *p* for trend < 0.001), with each 1‐SD increase associated with a 56% higher odds of GC (OR = 1.56, 95% CI: 1.33–1.85, *p* < 0.001). Subgroup analyses suggested that the inverse associations of PDI and hPDI with GC risk were more pronounced among smokers and individuals reporting low life stress, while the positive association of uPDI with GC risk appeared stronger in younger and married participants. Greater adherence to overall and healthy plant‐based diet patterns was associated with a lower risk of GC, whereas higher adherence to unhealthy plant‐based patterns was associated with a higher risk. These findings suggest that promoting higher‐quality plant‐based eating habits may be beneficial for GC prevention, particularly in high‐risk populations.

## Graphical abstract

This graphical abstract summarizes a sex‐matched case–control study conducted in high‐risk regions of Fujian Province, China, including 336 newly diagnosed gastric cancer cases and 336 community controls. Using a validated food frequency questionnaire, plant‐based diet quality was assessed by the overall Plant‐Based Diet Index (PDI), healthy Plant‐Based Diet Index (hPDI), and unhealthy Plant‐Based Diet Index (uPDI). Greater adherence to overall and healthy plant‐based dietary patterns was associated with a lower risk of gastric cancer, whereas higher adherence to an unhealthy plant‐based dietary pattern was associated with a significantly increased risk. Overall, the findings highlight the importance of plant‐based diet quality in gastric cancer prevention in high‐risk populations.

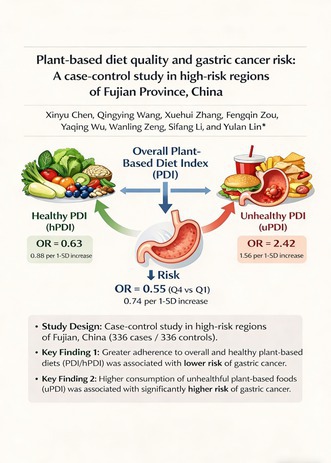

## 1 Introduction

Gastric cancer (GC) continues to represent a critical global health burden and remains one of the most lethal malignancies worldwide (Wang et al. 2024). The global burden of gastric cancer is projected to increase substantially by 2050 (Wang et al. 2024). China contributes disproportionately to this burden, accounting for close to 40% of global cases and deaths (Bray et al. 2024). Within the country, southeastern provinces such as Fujian are recognized as high‐incidence regions, where GC ranks among the top five cancers in both morbidity and mortality (Chen et al. 2023).

In recent years, increasing attention has been directed toward plant‐based diets because of their potential role in the prevention of chronic disease and cancer, including digestive system cancers (Zhao et al. 2022). Importantly, not all plant‐derived foods exert equal health effects. Nutrient‐dense options such as fruits, vegetables, and whole grains are rich in fiber and antioxidants, which may help reduce chronic inflammation and support a healthier gut microbiota, thereby contributing to anticancer processes. In contrast, refined grains, sugar‐sweetened beverages, and desserts—typically low in fiber and high in added sugars—may promote carcinogenesis, potentially through pro‐inflammatory pathways and microbiota dysbiosis (Satija et al. 2017; Elizabeth et al. 2020). This heterogeneity highlights the need to distinguish the quality of plant‐based diets when evaluating their health impacts.

Preventive approaches are essential given the multifactorial etiology of GC (Rebbeck et al. 2018; Chen et al. 2018; Malfertheiner et al. 2012). Among these, dietary modification stands out as a practical and modifiable factor. Compared with nutrient‐specific analyses, dietary pattern approaches better capture real‐world eating behaviors and account for potential synergistic or antagonistic effects across food groups (Abd Rashid et al. 2023; Wu et al. 2023). To operationalize this framework, researchers have established the plant‐based diet index (PDI) and its two derivatives: the healthy PDI (hPDI), emphasizing beneficial plant foods, and the unhealthy PDI (uPDI), which reflects diets dominated by less healthful plant‐based items (Satija et al. 2017).

Accumulating epidemiological evidence has linked adherence to plant‐based dietary patterns with reduced all‐cause mortality (Chen et al. 2022; Baden et al. 2019; Kim et al. 2021) and lower risks of several cancers, including colorectal (Loeb et al. 2022), breast (Rigi et al. 2021), prostate (Liu et al. 2024), and other gastrointestinal malignancies (Cai et al. 2024; Silva et al. 2024). Nevertheless, data specifically examining the relationship between plant‐based dietary indices (PDI/hPDI/uPDI) and GC risk remain limited, with only a small number of observational studies available to date (Rosenfeld et al. 2023).

Fujian Province, located in southeastern China, is recognized as a high‐risk region for GC, with a higher incidence than the national average (33.1/100,000 vs. 30.0/100,000) (Minggang and Xiongwei 2018). Moreover, some areas within Fujian have reported markedly higher GC mortality than the national average (49.47/100,000 vs. 21.9/100,000) (Li et al. 2017; Chen et al. 2019). This elevated burden may be partly related to local dietary habits, including relatively high consumption of salted and preserved foods, which have been linked to increased GC risk in prior studies (Tsugane 2005). We hypothesized that higher adherence to overall and healthy plant‐based diet patterns (PDI and hPDI) would be inversely associated with GC risk, whereas higher adherence to unhealthy plant‐based patterns (uPDI) would be positively associated with risk. Therefore, by differentiating between healthy and unhealthy plant‐based foods, we aimed to clarify their distinct associations with GC risk and to inform region‐specific dietary strategies for primary prevention.

## 2 Methods

### 2.1 Study Design and Study Participants

This investigation adopted a sex‐matched case–control design (with a 1:1 ratio) in Fujian Province, China. Eligible participants were between 18 and 75 years of age and had resided locally for at least 6 months within the year preceding enrollment. All individuals were required to communicate effectively and to provide written informed consent before participation.

The case group comprised patients newly diagnosed with GC at Fujian Medical University Union Hospital between July 2023 and November 2024. Diagnosis was verified by histopathological or cytological examination. Exclusion criteria included a previous history of malignant tumors, pregnancy or breastfeeding, and implausible daily caloric intake values (< 500 or > 3600 kcal for females; < 600 or > 4200 kcal for males) (Willett 2013).

The control group consisted of healthy community residents recruited during the same period from nine prefecture‐level cities within Fujian Province. Controls were matched to cases by sex. Sex was selected as the matching factor because it is a major determinant of dietary behaviors and social roles in this population. In contrast, age matching was not implemented due to feasibility constraints during community‐based recruitment: identifying an eligible control within a narrow age range for each newly diagnosed case (while meeting all inclusion/exclusion criteria and completing the FFQ interview) was operationally challenging within the study period. To minimize residual confounding by age, age information was collected for all participants and was adjusted for in all multivariable models; additional age‐stratified analyses were also conducted. Individuals were excluded if they had any past or current malignancy, severe medical conditions such as stroke or psychiatric disorders, or reported implausible energy intakes as defined above.

### 2.2 Data Collection

This study collected general demographic characteristics of participants through face‐to‐face interviews, including age, sex, marital status, education level, occupation, monthly household income per capita, smoking, alcohol consumption, daily life stress level, etc. Dietary intake was assessed using a previously validated 78‐item semi‐quantitative FFQ (Cheng et al. 2025). Clinical data were obtained through the hospital medical records system, including diagnostic information, date of diagnosis, body mass index (BMI), and other relevant clinical indicators. This study was approved by the Ethics Committee of Fujian Medical University (FJMU No. 2020[53]).

#### 2.2.1 Food Frequency Questionnaire (FFQ)

A structured, semi‐quantitative FFQ was used to assess dietary intake. Participants were instructed to report their habitual dietary intake over the preceding 12 months. The FFQ was administered in person at enrollment. Standardized instructions were provided by study staff, and questionnaires were checked for completeness at the time of collection. The FFQ covered 78 individual food items or food groups across 13 major categories, including: staple foods (8 items), root vegetables (3 items), pickled/grilled/fried foods (4 items), eggs (2 items), fresh meats (5 items), seafood (5 items), dairy products (4 items), snacks and nuts (4 items), beverages (3 items), soy products (6 items), fresh vegetables (17 items), fresh fruits (12 items), and dried foods (5 items).

The reliability and validity of this FFQ have been evaluated in adults from Fujian, China, using a 1‐month test–retest design (FFQ‐1 vs. FFQ‐2) and a 3‐day 24‐h dietary recall (3d‐24HDR) as the reference method (Cheng et al. 2025). For reliability, Spearman correlation coefficients ranged from 0.60 to 0.80 for food groups and from 0.66 to 0.96 for energy and nutrient intake, with intraclass correlation coefficients (ICCs) ranging from 0.53 to 0.91 and from 0.57 to 0.97, respectively. For relative validity against the 3d‐24HDR, Spearman correlations ranged from 0.41 to 0.72 for food groups and from 0.40 to 0.70 for nutrients. The proportion of participants classified into the same or adjacent tertile was 78.8%–95.1%, with fewer than 15% misclassified into distant categories, indicating acceptable agreement.

To evaluate plant‐based dietary patterns, these items were aggregated into 14 predefined food groups, classified as either plant‐based or animal‐based. The plant‐based groups comprised whole grains, fresh vegetables, fresh fruits, nuts, legumes, fruit juices, sugar‐sweetened beverages, refined grains, and desserts. The animal‐based groups included meat, dairy products, seafood, eggs, and animal offal.

Participants reported their usual consumption frequency over the preceding year. The original response options provided nine categories: (1) ≥ 4 times per day; (2) 2–3 times per day; (3) once per day; (4) 4–6 times per week; (5) 2–3 times per week; (6) once per week; (7) 1–3 times per month; (8) occasionally; (9) never. For analysis, these responses were subsequently collapsed into five broader frequency categories: more than once daily, once daily, 4–6 times weekly, 1–3 times weekly, and rarely/never.

#### 2.2.2 Demographics and Lifestyles

In addition to dietary information, data on sociodemographic characteristics and lifestyle behaviors were collected. Demographic variables included age, sex, education, occupation, household income, and perceived life stress levels in daily life. Height and weight were self‐reported, and body mass index (BMI) was calculated as weight (kg) divided by height squared (m^2^).

Lifestyle variables covered smoking and alcohol use during the past 12 months. Individuals were classified as current smokers if they had smoked regularly (at least one cigarette per day for six consecutive months or a cumulative total of ≥ 150 cigarettes). Regular alcohol consumers were defined as those who consumed alcohol at least once per week for a minimum of 6 months. Participants who did not meet these thresholds were regarded as non‐smokers or non‐drinkers.

### 2.3 Nutrients Calculation

The reported consumption frequency and usual portion size for each FFQ item were converted into average daily intake (g/day or mL/day) using the following equation: daily intake = frequency × portion size ÷ number of days in the corresponding time period. Daily nutrient intakes were then estimated by multiplying the daily amount of each food item by its nutrient composition and summing the values across all items. Nutrient intakes derived from the FFQ were calculated using the Nutrition Calculation Software (version 2.8.0(k)) developed by the National Institute for Nutrition and Food Safety, Chinese Center for Disease Control and Prevention. Nutrient values were matched to the Chinese Food Composition Table (6th Edition).

### 2.4 Dietary Index Calculation

In this study, plant‐based foods were further categorized into healthy and unhealthy plant‐based foods. Healthy plant‐based foods included whole grains, fresh vegetables, fresh fruits, nuts, and legumes, while unhealthy plant‐based foods comprised fruit juices, sugar‐sweetened beverages, refined grains, and desserts. Consistent with Satija et al. (2016), fruit juice was classified as a less‐healthy plant food; moreover, the FFQ item “fruit juice” did not distinguish between 100% juice and sweetened juice drinks, which are common in China, and therefore we retained the standard classification and noted this contextual heterogeneity when interpreting the indices.

Following previous studies (Satija et al. 2016; Wang et al. 2023), standardized scoring methods were applied to quantify the intake frequency of each food group. Most food groups were divided into five categories according to intake frequency, with scores assigned from 5 to 1 (“> 1 serving/day” = 5 points; “1 serving/day” = 4 points; “4–6 servings/week” = 3 points; “1–3 servings/week” = 2 points; “rarely or never” = 1 point). For fruits and vegetables, intake was first converted into average daily intake and then divided into quintiles, which were assigned scores from 5 to 1. The scores of all 14 food groups were then summed to construct the composite dietary indices.

In the construction of specific indices, plant‐based foods were scored positively, with higher intake assigned higher scores (from 1 to 5), whereas animal‐based foods were reverse scored, with higher intake assigned lower scores (from 5 to 1). Thus, a higher PDI reflects greater consumption of plant‐based foods and lower consumption of animal‐based foods. For hPDI, healthy plant‐based foods were positively scored, whereas unhealthy plant‐based foods and animal‐based foods were reverse scored. In contrast, for uPDI, unhealthy plant‐based foods were positively scored, while healthy plant‐based foods and animal‐based foods were reverse scored. This differentiated scoring approach allows for the effective distinction among overall, healthy, and unhealthy plant‐based dietary patterns, thereby enabling a comprehensive assessment of their potential associations with GC risk.

### 2.5 Sample Size and Power Calculation

The minimum sample size for this 1:1 sex‐matched case–control study was estimated using the standard formula for matched case–control designs. Assuming a two‐sided α of 0.05% and 90% power, an exposure prevalence among controls of *P*_0_ = 0.33, and an anticipated odds ratio (OR) of 1.77, the exposure prevalence among cases was calculated as *P*_1_ = OR × *P*_0_/(1 − *P*_0_ + OR × *P*_0_). The required number of matched pairs was then obtained using the following formula: *m* = (*Z*_α/2_ + *Z*_β_)^2^ × [*P*_0_ (1 − *P*_1_) + *P*_1_ (1 − *P*_0_)]/(*P*_1_ − *P*_0_)^2^, yielding approximately 279 matched pairs. We finally recruited 336 matched pairs. Assuming a two‐sided *α* = 0.05, a 1:1 case–control ratio (*n* = 336 per group), a control exposure prevalence of 50%, and a hypothesized OR of 0.60, the study provided approximately 90% power (about 91%) to detect this effect (with a two‐sided test). This suggests that the study had adequate statistical power for the primary association of interest.

### 2.6 Statistical Analysis

Continuous variables were summarized as means with standard deviations (SD) when normally distributed, or as medians with interquartile ranges (IQRs) otherwise. Categorical variables were expressed as counts and percentages. Group differences were examined using the chi‐square test for categorical variables and either the *t*‐test or one‐way ANOVA for continuous data, depending on the distribution of the data.

To control for total energy intake, nutrient values were energy‐adjusted using the residual method. Specifically, each nutrient was regressed on total energy intake, and the residuals were added to the expected nutrient intake at the mean energy intake to obtain energy‐adjusted nutrient values. Energy‐adjusted nutrient intakes were then compared between cases and controls. Participants were classified into quartiles of PDI, hPDI, and uPDI based on the distribution observed in the control group. Odds ratios (ORs) and 95% confidence intervals (CIs) for GC across quartiles were estimated using conditional logistic regression to account for the sex‐matched design. In multivariable models, we adjusted for key potential confounders selected a priori and/or identified from baseline comparisons, including age group (≤ 55 vs. > 55 years), marital status (married vs. single/separated/divorced/widowed), smoking status (yes/no), and daily life stress (none/low vs. moderate/high). To flexibly model the potential non‐linear dose–response relationships between the plant‐based dietary indices (PDI, hPDI, uPDI) and gastric cancer risk, we employed restricted cubic spline (RCS) regression with four knots placed at the 5th, 35th, 65th, and 95th percentiles of each dietary index distribution. The median value of each dietary index was used as the reference. All models were adjusted for the same set of covariates as those used in the primary analyses. A sensitivity analysis with further adjustment for family history of cancer was performed.

Further subgroup analyses were conducted to examine whether the associations between the dietary indices and GC risk varied across different strata. Stratification variables were selected based on their baseline differences between cases and controls and their established or plausible relevance to GC susceptibility and dietary behaviors (Rota et al. 2024; Collatuzzo et al. 2023). Subgroup analyses were considered exploratory, and effect modification was evaluated by including interaction terms between each dietary index and the stratification variable in conditional logistic regression models (using the Wald test).

All analyses were performed using SPSS version 26.0 (IBM Corp., Armonk, NY, USA), and all tests were two‐sided, with *p* < 0.05 considered statistically significant.

## 3 Results

### 3.1 Baseline Demographics

As shown in Table 1, compared with controls, GC cases were significantly older (56.79 ± 10.34 vs. 53.86 ± 11.13 years, *p* < 0.001), more likely to be married (93.8% vs. 88.1%, *p* = 0.011), and more likely to smoke (39.0% vs. 30.4%, *p* = 0.019). No significant differences were observed in sex, education, occupation, or income. Regarding dietary indices, cases had lower PDI (34.91 ± 3.54 vs. 36.09 ± 3.14, *p* < 0.001) and hPDI (49.49 ± 3.44 vs. 50.00 ± 3.20, *p* = 0.046) scores, but a higher uPDI (44.84 ± 4.04 vs. 43.12 ± 3.79, *p* < 0.001) than controls.

**TABLE 1:** Comparison of general demographic information of the study population (*N* = 672).

Variables	Total (*N* = 672)	Cases (*N* = 336)	Controls (*N* = 336)	*p*
Age, years, mean ± SD	55.31 ± 10.83	56.76 ± 10.34	53.86 ± 11.13	< 0.001
Age group, years				
≤ 55	320 (47.6)	132 (39.3)	188 (56.0)	< 0.001
> 55	352 (52.4)	204 (60.7)	148 (44.0)	
Sex				1.000
Male	380 (56.5)	190 (56.5)	190 (56.5)	
Female	292 (43.5)	146 (43.5)	146 (43.5)	
BMI (kg/m^2^)				0.068
< 24	415 (61.8)	196 (58.3)	219 (65.2)	
≥ 24	257 (38.2)	140 (41.7)	117 (34.8)	
Marital status				0.011
Married	611 (90.9)	315 (93.8)	296 (88.1)	
Single/separated/divorced/widowed	61 (9.1)	21 (6.2)	40 (11.9)	
Education level				0.157
Primary school or below	276 (41.1)	143 (42.6)	133 (39.6)	
Secondary school	177 (26.3)	90 (26.8)	87 (25.9)	
High school	105 (15.6)	58 (17.3)	47 (14.0)	
College	51 (7.6)	20 (5.9)	31 (9.2)	
University or above	63 (9.4)	25 (7.4)	38 (11.3)	
Occupation				0.361
Farmers/manual workers	199 (29.6)	99 (29.5)	100 (29.8)	
Other occupations	239 (35.6)	112 (33.3)	127 (37.8)	
Homemakers/retired/unemployed	234 (34.8)	125 (37.2)	109 (32.4)	
Average monthly household income, RMB				0.167
< 3000	64 (9.5)	26 (7.7)	38 (11.3)	
3000–6000	229 (34.1)	123 (36.6)	106 (31.6)	
> 6000	379 (56.4)	187 (55.7)	192 (57.1)	
Smoking				0.019
Yes	233 (34.7)	131 (39.0)	102 (30.4)	
No	439 (65.3)	205 (61.0)	234 (69.6)	
Alcohol drinking				0.916
Yes	107 (15.9)	53 (15.8)	54 (16.1)	
No	565 (84.1)	283 (84.2)	282 (83.9)	
Daily life stress				< 0.001
None/low	409 (60.9)	227 (67.6)	182 (54.2)	
Moderate/high	263 (39.1)	109 (32.4)	154 (48.8)	
Family history of cancer				< 0.001
Yes	129 (19.2)	82 (24.4)	47 (14.0)	
No	430 (64.0)	244 (72.6)	186 (55.4)	
Missing	113 (16.8)	10 (3.0)	103 (30.6)	
PDI, mean ± SD	35.49 ± 3.40	34.91 ± 3.54	36.09 ± 3.14	< 0.001
hPDI, mean ± SD	49.75 ± 3.33	49.49 ± 3.44	50.00 ± 3.20	0.046
uPDI, mean ± SD	43.98 ± 4.01	44.84 ± 4.04	43.12 ± 3.79	< 0.001

### 3.2 Comparison of Energy‐Adjusted Dietary Nutrient Intakes

As shown in Table 2, the intakes of 24 energy‐adjusted nutrients were compared between cases and controls. Compared with controls, GC cases had lower carbohydrate intake (96.10 ± 18.87 vs. 100.71 ± 19.70 g/day, *p* = 0.002). With respect to fat‐related nutrients, cases reported higher intakes of saturated fatty acids (7.23 ± 1.85 vs. 6.50 ± 1.87 g/day, *p* < 0.001), monounsaturated fatty acids (7.42 ± 1.72 vs. 6.86 ± 1.79 g/day, *p* < 0.001), and polyunsaturated fatty acids (3.38 ± 0.83 vs. 3.20 ± 0.90 g/day, *p* = 0.006). In addition, vitamin D and copper intakes intake was lower among cases than among controls (0.88 ± 0.42 vs. 0.95 ± 0.38 μg/day, *p* = 0.039; 0.68 ± 0.28 vs. 0.75 ± 0.31 mg/day, *p* = 0.002, respectively), whereas selenium was higher among cases (22.88 ± 14.14 vs. 20.77 ± 9.60 μg/day, *p* = 0.024). No statistically significant differences were observed for the remaining nutrients.

**TABLE 2:** Energy‐adjusted dietary nutrient intakes of study participants.

Nutrients	Overall (*N* = 672)	Cases (*N* = 336)	Controls (*N* = 336)	*p*
Protein (g)	23.79 ± 5.92	24.05 ± 6.10	23.53 ± 5.72	0.263
Carbohydrates (g)	98.42 ± 19.42	96.10 ± 18.87	100.71 ± 19.70	0.002
Fat (g)	11.27 ± 4.06	11.36 ± 3.75	11.17 ± 4.35	0.541
Saturated fatty acids (g)	6.86 ± 1.89	7.23 ± 1.85	6.50 ± 1.87	< 0.001
Monounsaturated fatty acids (g)	7.14 ± 1.78	7.42 ± 1.72	6.86 ± 1.79	< 0.001
Polyunsaturated fatty acids (g)	3.29 ± 0.88	3.38 ± 0.83	3.20 ± 0.90	0.006
Cholesterol (mg)	328.18 ± 131.60	322.48 ± 139.19	333.80 ± 123.63	0.267
Dietary fiber (g)	7.02 ± 4.04	6.84 ± 4.16	7.20 ± 3.90	0.251
Folate (μg)	113.02 ± 68.38	113.98 ± 68.72	112.07 ± 68.14	0.718
Vitamin A (μgRE)	227.90 ± 111.39	227.44 ± 109.65	228.35 ± 113.24	0.916
Vitamin B1 (mg)	0.25 ± 0.09	0.24 ± 0.08	0.26 ± 0.09	0.057
Vitamin B2 (mg)	0.35 ± 0.12	0.34 ± 0.13	0.36 ± 0.11	0.052
Vitamin B3 (mg)	2.90 ± 1.43	2.92 ± 1.57	2.88 ± 1.32	0.684
Vitamin B6 (mg)	0.12 ± 0.14	0.13 ± 0.14	0.12 ± 0.13	0.195
Vitamin C (mg)	85.21 ± 55.55	83.17 ± 54.10	87.23 ± 56.95	0.345
Vitamin D (μg)	0.91 ± 0.40	0.88 ± 0.42	0.95 ± 0.38	0.039
Vitamin E (mg)	5.81 ± 1.79	5.73 ± 1.55	5.88 ± 2.00	0.295
β‐carotene (μg)	4665.72 ± 2979.94	4782.31 ± 3010.64	4550.85 ± 2949.35	0.316
Iron (mg)	6.12 ± 2.64	6.30 ± 3.02	5.95 ± 2.21	0.091
Zinc (mg)	3.01 ± 1.05	3.06 ± 1.09	2.96 ± 1.02	0.196
Magnesium (mg)	111.49 ± 39.97	111.23 ± 41.43	111.75 ± 38.54	0.866
Selenium (μg)	21.82 ± 12.10	22.88 ± 14.14	20.77 ± 9.60	0.024
Calcium (mg)	192.70 ± 84.59	197.67 ± 86.49	187.80 ± 82.51	0.132
Copper (mg)	0.71 ± 0.30	0.68 ± 0.28	0.75 ± 0.31	0.002

### 3.3 Plant‐Based Diets and GC Risk

**FIGURE 1:**
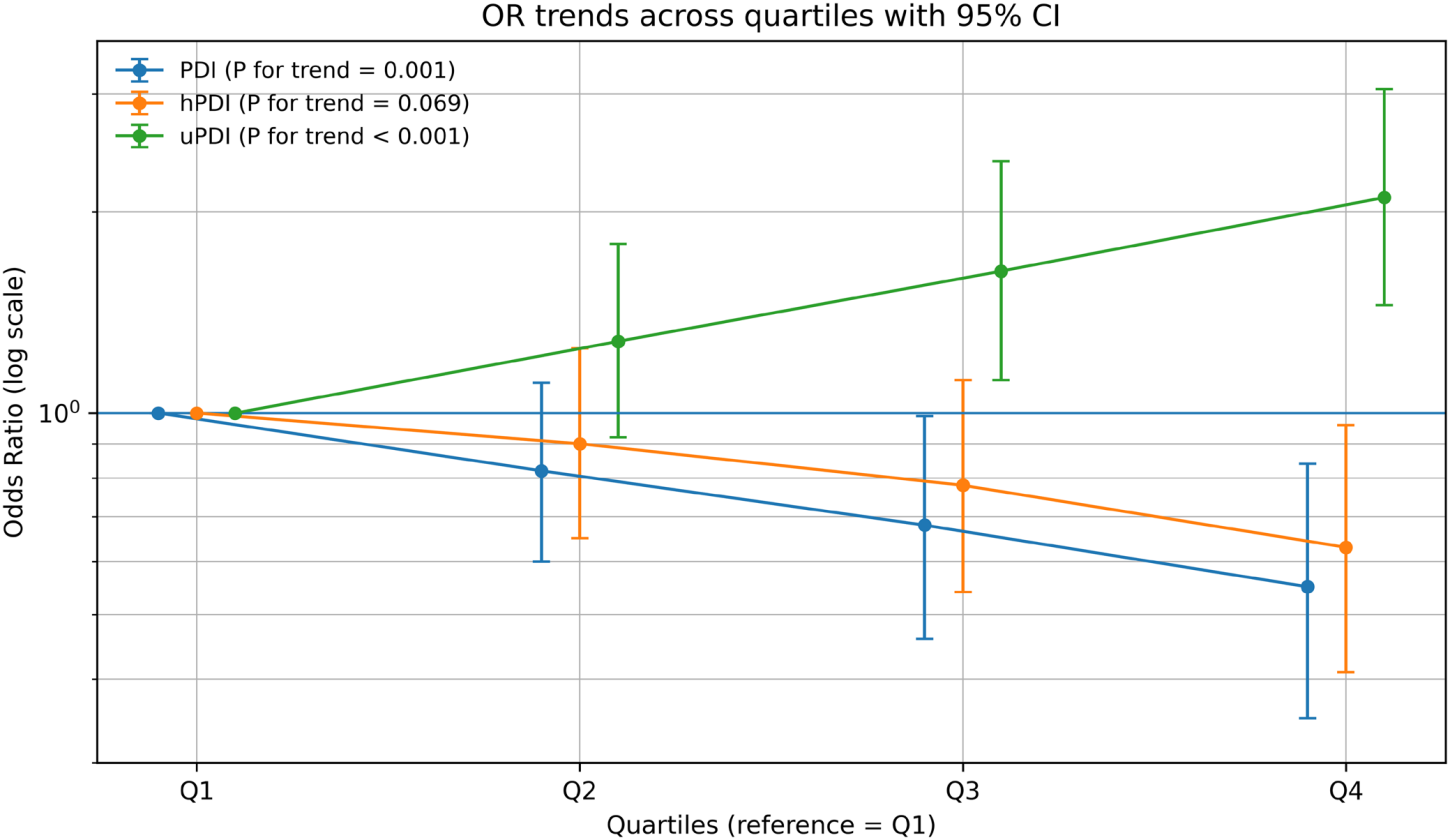
Odds ratios (ORs) and 95% confidence intervals for gastric cancer across quartiles of plant‐based diet indices (PDI, hPDI, and uPDI).

As shown in Table 3 and Figure 1, different types of plant‐based dietary patterns were significantly associated with GC risk. Higher PDI scores were inversely associated with GC risk. Multivariable logistic regression showed that participants in the highest PDI quartile (Q4) had a 45% lower odds of GC compared with those in the lowest quartile (Q1) (OR = 0.55, 95% CI: 0.35–0.84, *p* = 0.006), with a clear dose–response trend (*p* for trend = 0.001). Each 1‐SD increase in PDI was associated with a 26% lower odds of GC (OR = 0.74, 95% CI: 0.63–0.87, *p* < 0.001). Similarly, hPDI was negatively associated with GC risk. Participants in Q4 had a 37% lower odds of GC compared with Q1 (OR = 0.63, 95% CI: 0.41–0.96, *p* = 0.033), although the linear trend across quartiles did not reach statistical significance (*p* for trend = 0.069). In contrast, uPDI showed a strong positive association with GC risk. Individuals in Q4 had a 2.42‐fold higher odds of GC compared with Q1 (OR = 2.42, 95% CI: 1.55–3.78, *p* < 0.001), with a significant dose–response trend (*p* for trend < 0.001). Each 1‐SD increase in uPDI was associated with a 56% higher odds of GC (OR = 1.56, 95% CI: 1.33–1.85, *p* < 0.001). In sensitivity analyses with additional adjustment for family history of cancer, the associations between PDI/hPDI/uPDI and GC risk were materially unchanged (Table S1).

**TABLE 3:** Odds ratios (ORs) and 95% confidence intervals (CIs) for gastric cancer across quartiles of PDI, hPDI, and uPDI.

Index	Case/control	OR^1^ (95%)	*p*	OR^2^ (95%)	*p*
PDI	336/336				
Quartile 1	112/71	Reference		Reference	
Quartile 2	83/71	0.74 (0.48–1.14)	0.176	0.78 (0.50–1.22)	0.782
Quartile 3	62/91	0.43 (0.28–0.67)	< 0.001	0.46 (0.30–0.73)	< 0.001
Quartile 4	79/103	0.49 (0.32–0.73)	< 0.001	0.55 (0.35–0.84)	0.006
*p* for trend			< 0.001		0.001
Continuous (per SD increase)		0.70 (0.60–0.82)	< 0.001	0.74 (0.63–0.87)	< 0.001
hPDI	336/336				
Quartile 1	100/69	Reference		Reference	
Quartile 2	59/69	0.59 (0.37–0.94)	0.026	0.61 (0.38–0.98)	0.040
Quartile 3	87/93	0.65 (0.42–0.99)	0.043	0.71 (0.46–1.10)	0.126
Quartile 4	90/105	0.59 (0.39–0.90)	0.013	0.63 (0.41–0.96)	0.033
*p* for trend			0.025		0.069
Continuous (per 1 SD increase)		0.86 (0.74–0.99)	0.047	0.88 (0.75–1.03)	0.110
uPDI	336/336				
Quartile 1	55/84	Reference		Reference	
Quartile 2	38/63	0.92 (0.54–1.56)	0.760	0.92 (0.53–1.57)	0.748
Quartile 3	102/103	1.51 (0.98–2.34)	0.063	1.54 (0.98–2.41)	0.059
Quartile 4	141/86	2.50 (1.62–3.86)	< 0.001	2.42 (1.55–3.78)	< 0.001
*p* for trend			< 0.001		< 0.001
Continuous (per SD increase)		1.56 (1.33–1.84)	< 0.001	1.56 (1.33–1.85)	< 0.001

*Note:* OR^1^: unadjusted. OR^2^: adjusted to age group, marital status, daily life stress and smoking.

Restricted cubic spline (RCS) analyses were conducted to further assess potential non‐linear dose–response relationships between PDI/hPDI/uPDI and GC risk. As shown in Figures S1–S3, higher PDI and hPDI were generally associated with lower odds of GC, whereas higher uPDI was associated with higher odds of GC. Tests for non‐linearity did not indicate strong departures from linearity, suggesting that the observed associations were broadly monotonic across the exposure range (Figures S1–S3).

### 3.4 Stratified Analysis of Plant‐Based Diets and GC Risk

#### 3.4.1 Age

As shown in Table 4, stratified analyses suggested that the magnitude of the associations between plant‐based diet indices (PDI, hPDI, and uPDI) and GC risk varied by age group. Among participants aged > 55 years, higher PDI was inversely associated with GC risk (Q4 vs. Q1: OR = 0.52, 95% CI: 0.28–0.94, *p* = 0.030; *p* for trend = 0.007). In participants aged ≤ 55 years, a stronger inverse association was observed for hPDI (Q4 vs. Q1: OR = 0.43, 95% CI: 0.23–0.81, *p* = 0.009; *p* for trend = 0.019). For uPDI, the highest quartile was associated with higher GC risk in the ≤ 55‐year group (Q4 vs. Q1: OR = 3.43, 95% CI: 1.75–6.74; *p* for trend < 0.001), whereas in the > 55‐year group, uPDI showed a significant positive trend across quartiles, although the Q4 versus Q1 comparison did not reach statistical significance (Q4 vs. Q1: OR = 1.76, 95% CI: 0.96–3.22; *p* for trend = 0.021). No statistically significant interaction by age was detected for these indices (*p* for interaction > 0.05).

**TABLE 4:** Stratified multivariable logistic regression analysis of PDI, hPDI, and uPDI quartiles in relation to gastric cancer risk.

Subgroup	Cases/controls	Index	Q2 vs. Q1	Q3 vs. Q1	Q4 vs. Q1	*p* for trend	*p* for interaction
			OR (95% CI)^a^	OR (95% CI)^a^	OR (95% CI)^a^		
Age ≤ 55 years	132/188	PDI	0.89 (0.47–1.70)	0.64 (0.33–1.22)	0.58 (0.31–1.10)	0.060	0.740
		hPDI	0.54 (0.27–1.09)	0.66 (0.36–1.21)	0.43 (0.23–0.81)	0.019	0.116
		uPDI	1.02 (0.46–2.29)	1.72 (0.89–3.33)	3.43 (1.75–6.74)	< 0.001	0.135
Age > 55 years	204/148	PDI	0.70 (0.38–1.31)	0.35 (0.19–0.66)	0.52 (0.28–0.94)	0.007	—
		hPDI	0.68 (0.36–1.31)	0.79 (0.42–1.46)	0.86 (0.47–1.56)	0.778	—
		uPDI	0.78 (0.37–1.64)	1.32 (0.70–2.48)	1.76 (0.96–3.22)	0.021	—
Married	315/296	PDI	0.75 (0.47–1.20)	0.46 (0.29–0.74)	0.47 (0.30–0.75)	< 0.001	0.080
		hPDI	0.63 (0.38–1.04)	0.67 (0.42–1.05)	0.59 (0.37–0.92)	0.031	0.148
		uPDI	0.89 (0.51–1.55)	1.38 (0.87–2.19)	2.27 (1.43–3.60)	< 0.001	0.246
Single/separated/divorced/widowed	21/40	PDI	1.42 (0.30–6.76)	0.43 (0.07–2.76)	1.95 (0.48–7.97)	0.530	—
		hPDI	0.31 (0.04–2.22)	1.40 (0.30–6.46)	1.35 (0.28–6.58)	0.321	—
		uPDI	1.11 (0.05–23.03)	6.92 (0.70–68.27)	7.01 (0.73–67.51)	0.034	—
Smoker	131/102	PDI	0.46 (0.22–0.97)	0.32 (0.15–0.70)	0.22 (0.10–0.48)	< 0.001	0.017
		hPDI	0.33 (0.14–0.78)	0.33 (0.16–0.70)	0.26 (0.12–0.57)	< 0.001	0.010
		uPDI	0.75 (0.30–1.91)	1.61 (0.74–3.52)	3.23 (1.53–6.85)	< 0.001	0.236
Non‐smoker	205/234	PDI	1.04 (0.59–1.83)	0.58 (0.33–1.02)	0.84 (0.50–1.44)	0.256	—
		hPDI	0.82 (0.45–1.49)	1.10 (0.62–1.92)	0.96 (0.56–1.64)	0.877	—
		uPDI	1.00 (0.52–1.94)	1.45 (0.84–2.51)	2.04 (1.17–3.56)	0.005	—
Daily life stress at none/low level	227/182	PDI	0.64 (0.36–1.14)	0.32 (0.18–0.57)	0.30 (0.17–0.54)	< 0.001	0.001
		hPDI	0.45 (0.25–0.81)	0.63 (0.36–1.11)	0.47 (0.27–0.81)	0.023	0.253
		uPDI	0.97 (0.49–1.92)	1.58 (0.89–2.80)	2.30 (1.30–4.06)	0.001	0.696
Daily life stress at moderate/high level	109/154	PDI	1.14 (0.53–2.45)	0.92 (0.42–1.99)	1.34 (0.66–2.73)	0.496	—
		hPDI	0.99 (0.44–2.25)	0.90 (0.44–1.88)	0.96 (0.46–1.99)	0.872	—
		uPDI	0.82 (0.34–1.97)	1.37 (0.67–2.83)	2.61 (1.27–5.33)	0.003	—

^a^ Adjusted to age group, marital status, daily life stress, and smoking (excluding stratification factors).

#### 3.4.2 Marital Status

In married participants, higher PDI and hPDI were inversely associated with GC risk (PDI Q4 vs. Q1: OR = 0.47, 95% CI: 0.30–0.75; *p* = 0.001; *p* for trend < 0.001; hPDI Q4 vs. Q1: OR = 0.59, 95% CI: 0.37–0.92; *p* = 0.020; *p* for trend = 0.031), whereas higher uPDI was positively associated with GC risk (Q4 vs. Q1: OR = 2.27, 95% CI: 1.43–3.60; *p* < 0.001; *p* for trend < 0.001). In single/separated/divorced/widowed participants (21 cases/40 controls), the associations for PDI and hPDI were not statistically significant (PDI Q4 vs. Q1: OR = 1.95, 95% CI: 0.48–7.97; *p* for trend = 0.530; hPDI Q4 vs. Q1: OR = 1.35, 95% CI: 0.28–6.58; *p* for trend = 0.321), while uPDI showed a positive trend with wide confidence intervals (Q4 vs. Q1: OR = 7.01, 95% CI: 0.73–67.51; *p* for trend = 0.034). These estimates should be interpreted cautiously due to the small sample size in this subgroup. No statistically significant interaction by marital status was detected for these indices (*p* for interaction > 0.05).

#### 3.4.3 Smoking Status

Among smokers, the inverse associations for PDI and hPDI were more pronounced (PDI Q4 vs. Q1: OR = 0.22, 95% CI: 0.10–0.48; *p* < 0.001; *p* for trend < 0.001; hPDI Q4 vs. Q1: OR = 0.26, 95% CI: 0.12–0.57; *p* < 0.001; *p* for trend < 0.001). For uPDI, the association with higher GC risk was statistically significant among smokers (Q4 vs. Q1: OR = 2.75, 95% CI: 1.40–5.42; *p* for trend < 0.001), while estimates in non‐smokers were directionally similar but less precise (Q4 vs. Q1: OR = 2.03, 95% CI: 0.98–4.21; *p* for trend = 0.003). These differences by smoking status were supported by statistically significant interaction tests for PDI (*p* for interaction = 0.017) and hPDI (*p* for interaction = 0.010), whereas no significant interaction was observed for uPDI (*p* for interaction = 0.236).

#### 3.4.4 Life Stress

In participants reporting none/low daily life stress (227 cases/182 controls), higher PDI and hPDI were inversely associated with GC risk (PDI Q4 vs. Q1: OR = 0.30, 95% CI: 0.17–0.54; *p* for trend < 0.001; hPDI Q4 vs. Q1: OR = 0.47, 95% CI: 0.27–0.81; *p* for trend = 0.023). In contrast, among participants reporting moderate/high life stress (109 cases/154 controls), neither PDI nor hPDI showed statistically significant associations (PDI Q4 vs. Q1: OR = 1.34, 95% CI: 0.66–2.73; *p* for trend = 0.496; hPDI Q4 vs. Q1: OR = 0.96, 95% CI: 0.46–1.99; *p* for trend = 0.872). For uPDI, higher scores were positively associated with GC risk in both stress strata (none/low: OR = 2.30, 95% CI: 1.30–4.06; *p* for trend = 0.001; moderate/high: OR = 2.61, 95% CI: 1.27–5.33; *p* for trend = 0.003). The interaction test indicated effect modification by daily life stress for PDI (*p* for interaction = 0.001), while no significant interaction was detected for hPDI (*p* for interaction = 0.253) or uPDI (*p* for interaction = 0.696).

## 4 Discussion

In this case–control study conducted in a high‐incidence region of Fujian Province, southeastern China, we observed that greater adherence to overall and healthy plant‐based diets (PDI and hPDI) was associated with a lower risk of gastric cancer, whereas a higher unhealthy plant‐based diet index (uPDI) was associated with a higher risk. These associations persisted after multivariable adjustment. Overall, the findings underscore the importance of plant‐based diet quality in relation to GC susceptibility in high‐risk settings.

The inverse association observed for the PDI may be largely attributed to its bidirectional scoring approach, which positively weighs protective plant‐based foods—such as whole grains, vegetables, and fruits—known to be rich in dietary fiber, polyphenols, and antioxidant vitamins (Liu 2003; Gu et al. 2024), while penalizing animal‐based products, particularly processed meats and preserved seafood, which are sources of carcinogenic compounds including N‐nitroso compounds and excessive salt (Stuff et al. 2009). Compared with the seminal study by Satija et al. (2017) conducted in the U.S. population, the stronger protective association of PDI relative to hPDI observed in our study represents a divergence from patterns reported in Western cohorts. This discrepancy may reflect region‐specific dietary cultures and dominant dietary risk factors. In Western settings, major dietary risks often stem from unhealthy plant‐based foods, such as sugar‐sweetened beverages and refined desserts. In contrast, in southeastern China, animal‐based foods—particularly salted and preserved seafood—may contribute more prominently to GC risk than unhealthy plant‐based items.

Although hPDI was also inversely associated with GC risk, the effect size was smaller than that of PDI. One possible explanation for the weaker association observed for hPDI compared with PDI relates to the scoring scheme for certain plant foods. In hPDI, fruit juice is classified as an “unhealthy plant food” and is reverse‐scored; this classification follows the standard hPDI/uPDI framework, because fruit juice typically contains less fiber and may contribute more free sugars than whole fruit, and therefore its health effects may differ from those of whole fruits. However, 100% fruit juice may still provide fruit‐derived micronutrients and bioactive compounds (e.g., vitamin C and polyphenols), and an umbrella review has summarized evidence suggesting some potential health benefits of 100% juice consumption, although conclusions remain inconclusive (Beckett et al. 2025). Meanwhile, evidence from prospective syntheses is mixed and has also suggested a small positive association between 100% fruit juice intake and overall cancer risk, with generally low certainty (Pan et al. 2023). Taken together, this suggests that classifying all fruit juice uniformly as “unhealthy” may introduce some exposure misclassification and potentially attenuate the observed association for hPDI; further studies distinguishing between juice types (e.g., 100% juice vs. sweetened juice) and degree of processing are warranted. Some nutrients present in healthy plant foods (e.g., selenium) may exert non‐linear or threshold effects in cancer prevention (Weekley and Harris 2013; Roman et al. 2014). Conversely, higher uPDI scores were positively associated with GC risk, potentially reflecting the pro‐inflammatory and metabolically disruptive effects of refined grains, sugar‐sweetened beverages, and desserts (Kim et al. 2020; Gaesser 2020). Our findings regarding the harmful role of uPDI are consistent with previous studies. For instance, a large‐scale prospective cohort among UK adults reported that higher uPDI scores significantly increased the risks of cardiovascular disease (HR = 1.21, 95% CI: 1.05–1.20), cancer (HR = 1.10, 95% CI: 1.03–1.17), and all‐cause mortality (HR = 1.23, 95% CI: 1.14–1.32) (Thompson et al. 2023). Moreover, Srour et al. (2019) observed similar results in a large French cohort, where the adverse effects of unhealthy plant‐based foods were consistently evident across all population subgroups.

The nutrient comparisons provide additional context for the dietary index findings. Notably, the higher intakes of several fatty acid subtypes (SFA, MUFA, and PUFA) observed among cases are broadly consistent with the positive association between uPDI and GC risk, because uPDI captures dietary patterns that may involve greater consumption of energy‐dense, highly processed foods and cooking oils (e.g., refined grains, desserts, sugar‐sweetened beverages, and fried/processed items). Although total fat intake was not significantly different, these differences may reflect underlying food‐source patterns and food preparation methods rather than total fat quantity per se. Prior observational evidence suggests that higher intake of total fat and specific fat subtypes—particularly saturated fat—may be associated with GC risk, although findings across populations are not fully consistent (Han et al. 2015). In our study, these nutrient‐level contrasts are secondary and should be interpreted cautiously given the case–control design and FFQ‐based assessment; nevertheless, they are directionally consistent with the notion that diet quality and food sources (captured by PDI/hPDI/uPDI) may be relevant to GC susceptibility.

Stratified analyses further revealed population heterogeneity in these associations. Among older participants, PDI exerted stronger protective effects, possibly through improving gut microbiota balance and reducing carcinogen absorption in the context of age‐related declines in digestive function (Claesson et al. 2012). In younger participants, hPDI showed greater benefit, likely due to higher metabolic activity and more effective utilization of antioxidants such as vitamin C and polyphenols in neutralizing reactive oxygen species (Liguori et al. 2018). This finding is consistent with findings from a prospective cohort study on prostate cancer, which reported that higher intake of healthy plant‐based foods was significantly associated with a reduced risk of cancer among younger individuals (Loeb et al. 2022). Among married participants, the protective effects of PDI and hPDI were more pronounced, possibly explained by greater dietary regularity and a higher likelihood of home‐cooked meals with lower salt and fewer processed foods (Mills et al. 2017); however, uPDI also showed stronger harmful effects in this group, potentially due to shared household exposure to unhealthy plant‐based foods (Monteiro et al. 2018; Mills et al. 2018). In smokers, the protective associations of PDI and hPDI were particularly strong, as antioxidant nutrients such as vitamin E and β‐carotene may mitigate smoking‐induced oxidative stress and DNA damage (Menkes et al. 1986; Johansson et al. 2010; Kim et al. 2007). In participants with lower life stress, PDI and hPDI conferred stronger protection, possibly because a more stable physiological state facilitates the utilization of beneficial plant‐derived components (Lopresti 2020). Importantly, uPDI was consistently associated with an increased GC risk.

This study has several limitations. First, due to the case–control design and FFQ‐based dietary assessment, the findings may be subject to recall bias and measurement error, particularly if cases and controls reported past dietary habits differently. Second, reverse causation is possible in case–control settings: some participants with early or prodromal symptoms before diagnosis may have modified their diet (e.g., reducing meat or shifting toward “lighter” foods) prior to completing the FFQ, which could bias associations in either direction. Although we asked participants to report habitual intake over the preceding 12 months, this concern cannot be fully eliminated. Third, *Helicobacter pylori* status, an important risk factor for GC, was not available, and residual confounding cannot be fully excluded. In addition, cases and controls were not age‐matched and baseline age differed between groups; although age group was adjusted for in multivariable models, some residual confounding related to imperfect age balance may remain. Finally, the lack of detailed clinical information limited further analyses by histological subtype, reducing the granularity of the findings. Therefore, prospective studies with repeated dietary assessments and more complete clinical data are warranted to confirm these associations.

## 5 Implications and Conclusion

From a policy and prevention perspective, these findings support promoting high‐quality plant‐based eating patterns (similar to hPDI) in Fujian, where the burden of GC remains high. Region‐specific strategies could prioritize increasing intake of whole grains, vegetables, fruits, legumes, and nuts, while concurrently reducing salt intake and limiting salted/preserved foods—dietary practices common in coastal southeastern China and linked to GC risk. Practical implementation could include incorporating brief diet‐quality screening and counseling into community health services and high‐risk GC prevention programs, alongside broader measures such as salt‐reduction initiatives, healthy canteen standards, and culturally tailored public education. Importantly, emphasizing diet quality rather than simply encouraging greater consumption of plant food may help avoid inadvertently increasing intake of refined grains, sugary beverages, and desserts captured by uPDI. Overall, higher adherence to PDI and hPDI was associated with lower GC risk, whereas higher uPDI was associated with a higher risk; however, given the case–control design and FFQ‐based assessment, these associations should be interpreted cautiously and require prospective validation.

## Author Contributions

**Xinyu Chen:** conceptualization, formal analysis, methodology, software, writing – original draft, and writing – review and editing. **Qingying Wang:** formal analysis, methodology, and writing – review and editing. **Xuehui Zhang:** formal analysis, methodology, and writing – review and editing. **Fengqin Zou:** methodology, software, and writing – review and editing. **Yaqing Wu:** methodology and writing – review and editing. **Wanling Zeng:** data curation, methodology, and writing – review and editing. **Sifang Li:** data curation and writing – review and editing. **Yulan Lin:** conceptualization, data curation, methodology, project administration, supervision, and writing – review and editing.

## Funding

This work was supported by the National Natural Science Foundation of China (No. 72004025); Natural Science Foundation of Fujian Province, China (No. 2018J018); and Cultivation Program for Distinguished Young Scholar of Fujian Province University (No. 2017B020). The funder had no role in study design, data collection and analysis, decision to publish, or preparation of the manuscript.

## Ethics Statement

This study was conducted in accordance with the principles of the Declaration of Helsinki and was approved by the Ethics Committee of Fujian Medical University (FJMU No. 2020 [53]). Before participation, the purpose and content of the study were fully explained to the participants, and informed consent was obtained. Participants were free to withdraw from the study at any time without any consequences, and refusal to participate had no impact on their medical care. All personal information of the participants was kept strictly confidential throughout the study.

## Consent

Informed consent was obtained from all participants.

## Conflicts of Interest

The authors declare no conflicts of interest.

## Supporting information


**Table S1:** Sensitivity analysis: ORs (95% CIs) for gastric cancer across quartiles of PDI, hPDI, and uPDI after additional adjustment for family history of cancer.
**Figure S1:** Restricted cubic spline (RCS) plots for the correlations between PDI and gastric cancer risk.
**Figure S2:** Restricted cubic spline (RCS) plots for the correlations between hPDI and gastric cancer risk.
**Figure S3:** Restricted cubic spline (RCS) plots for the correlations between uPDI and gastric cancer risk.

## Data Availability

The data that support the findings of this study are available from the corresponding author, Yulan Lin, upon reasonable request. The data are not publicly available due to privacy and ethical restrictions.
