# Supplementary material for: Plant‐Based Diet Quality and Gastric Cancer Risk: A Case–Control Study in High‐Risk Regions of Fujian Province, China
Source: Food Sci Nutr. 2026 Apr 13;14(4):e71756. doi: 10.1002/fsn3.71756 (PMC13071758; doi:10.1002/fsn3.71756)
Supplement: Supplementary file 1 — Table S1: Sensitivity analysis: ORs (95% CIs) for gastric cancer across quartiles of PDI, hPDI, and uPDI after additional adjustment for family history of cancer. Figure S1: Restricted cubic spline (RCS) plots for the correlations between PDI and gastric cancer risk. Figure S2: Restricted cubic spline (RCS) plots for the correlations between hPDI and gastric cancer risk. Figure S3: Restricted cubic spline (RCS) plots for the correlations between uPDI and gastric cancer risk. [file FSN3-14-e71756-s001.docx]

**Supplementary Table S1.** Sensitivity analysis: ORs (95% CIs) for gastric cancer across quartiles of PDI, hPDI, and uPDI after additional adjustment for family history of cancer.

| Index | Case/control | OR^1^ (95%) | *P* value | OR^2^ (95%) | *P* value |
| --- | --- | --- | --- | --- | --- |
| PDI | 336 / 336 |  |  |  |  |
| Quartile 1 | 112 / 71 | Reference |  | Reference |  |
| Quartile 2 | 83 / 71 | 0.74 (0.48-1.14) | 0.176 | 0.88 (0.54-1.44) | 0.618 |
| Quartile 3 | 62 / 91 | 0.43 (0.28-0.67) | <0.001 | 0.52 (0.32-0.86) | 0.010 |
| Quartile 4 | 79 / 103 | 0.49 (0.32-0.73) | <0.001 | 0.61 (0.38-0.98) | 0.041 |
| *P* for trend |  |  | <0.001 |  | 0.011 |
| Continuous (per SD increase) |  | 0.70 (0.60-0.82) | <0.001 | 0.77 (0.65-0.92) | 0.004 |
| hPDI | 336 / 336 |  |  |  |  |
| Quartile 1 | 100 / 69 | Reference |  | Reference |  |
| Quartile 2 | 59 / 69 | 0.59 (0.37-0.94) | 0.026 | 0.52 (0.30-0.88) | 0.015 |
| Quartile 3 | 87 / 93 | 0.65 (0.42-0.99) | 0.043 | 0.67 (0.41-1.09) | 0.104 |
| Quartile 4 | 90 / 105 | 0.59 (0.39-0.90) | 0.013 | 0.60 (0.37-0.98) | 0.042 |
| *P* for trend |  |  | 0.025 |  | 0.110 |
| Continuous (per 1 SD increase) |  | 0.86 (0.74-0.99) | 0.047 | 0.88 (0.74-1.05) | 0.151 |
| uPDI | 336 / 336 |  |  |  |  |
| Quartile 1 | 55 / 84 | Reference |  | Reference |  |
| Quartile 2 | 38 / 63 | 0.92 (0.54-1.56) | 0.760 | 1.13 (0.63-2.04) | 0.676 |
| Quartile 3 | 102 / 103 | 1.51 (0.98-2.34) | 0.063 | 1.61 (0.99-2.62) | 0.053 |
| Quartile 4 | 141 / 86 | 2.50 (1.62-3.86) | <0.001 | 2.55 (1.57-4.14) | <0.001 |
| *P* for trend |  |  | <0.001 |  | <0.001 |
| Continuous (per SD increase) |  | 1.56 (1.33-1.84) | <0.001 | 1.55 (1.30-1.86) | <0.001 |

OR^1^: Unadjusted

OR^2^: Adjusted to age group, marital status, daily life stress, smoking and family history of cancer.


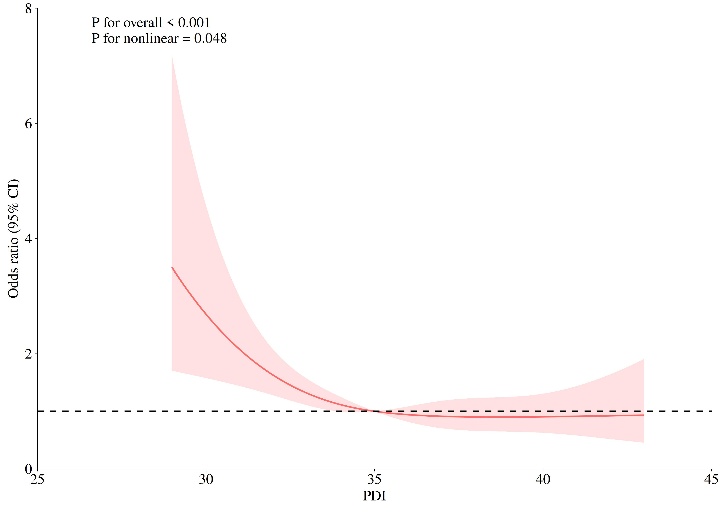


**Supplementary Figure S1**. Restricted cubic spline (RCS) plots for the correlations between PDI and gastric cancer risk


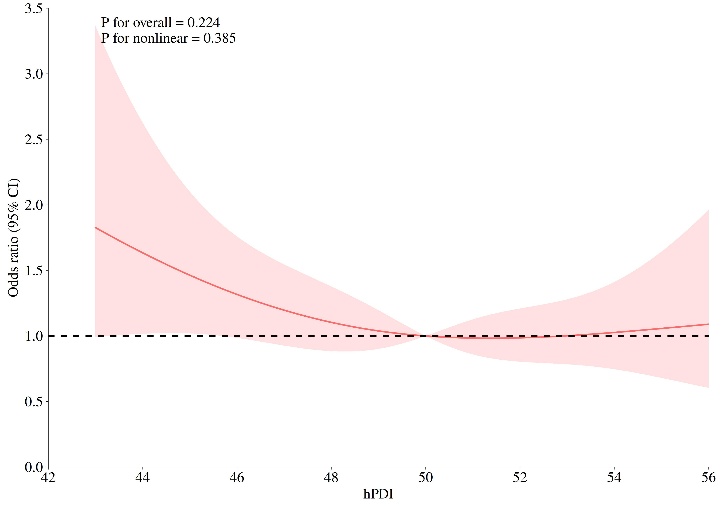


**Supplementary Figure S2**. Restricted cubic spline (RCS) plots for the correlations between hPDI and gastric cancer risk


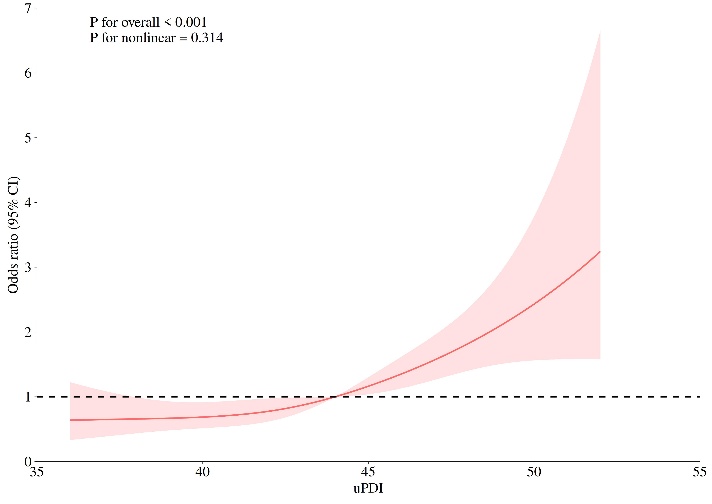


**Supplementary Figure S3**. Restricted cubic spline (RCS) plots for the correlations between uPDI and gastric cancer risk
